# Hexa­kis­(thio­urea-κ*S*)nickel(II) nitrate: a redetermination

**DOI:** 10.1107/S1600536810026668

**Published:** 2010-07-10

**Authors:** Muhammad Monim-ul-Mehboob, Mehmet Akkurt, Islam Ullah Khan, Shahzad Sharif, Iram Asif, Saeed Ahmad

**Affiliations:** a Department of Chemistry, University of Engineering and Technology, Lahore 54890, Pakistan; b Department of Physics, Faculty of Arts and Sciences, Erciyes University, 38039 Kayseri, Turkey; c Materials Chemistry Laboratory, Department of Chemistry, Government College University, Lahore 54000, Pakistan

## Abstract

A preliminary X-ray study of the title mol­ecular salt, [Ni(CH_4_N_2_S)_6_](NO_3_)_2_, has been reported twice previously, by Maďar [*Acta Cryst.* (1961), **14**, 894] and Rodriguez, Cubero, Vega, Morente & Vazquez [*Acta Cryst.* (1961), **14**, 1101], using film methods. We confirm the previous studies, but to modern standards of precision and with all H atoms located. The central Ni atom (site symmetry 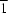) of the dication is octa­hedrally coordinated by six S-bound thio­urea mol­ecules. The crystal structure is stabilized by intra- and inter­molecular N—H⋯S and N—H⋯O hydrogen bonds.

## Related literature

The structure of the title complex at room temperature has been reported twice previously, see: Maďar (1961 ▶); Rodriguez *et al.* (1961 ▶). For the biological and non-linear optical properties and applications of metal complexes of thio­urea-type ligands, see: Arslan *et al.* (2009 ▶); Emre *et al.* (2009 ▶); Bhaskaran *et al.* (2007 ▶); Eaton & Law(1975 ▶); Figgis & Reynolds (1986 ▶). For the crystal structures of some similar Ni complexes, see: Suescun *et al.* (2000 ▶); Zhu *et al.* (2009 ▶). For reference structural data, see: Allen *et al.* (1987 ▶).
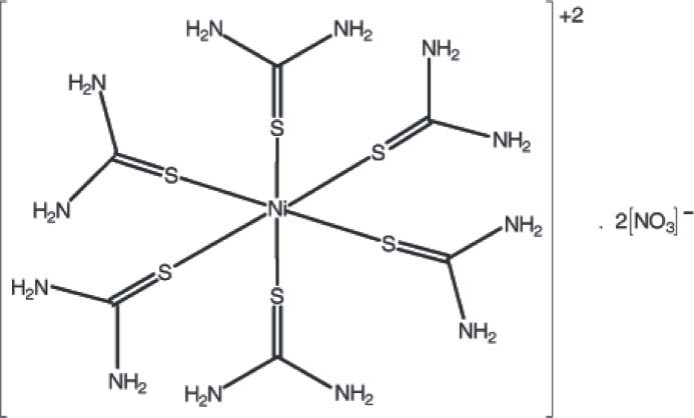


## Experimental

### 

#### Crystal data

[Ni(CH_4_N_2_S)_6_](NO_3_)_2_
*M* *_r_* = 639.50
Monoclinic, 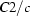

*a* = 22.4433 (6) Å
*b* = 9.2398 (3) Å
*c* = 16.3136 (5) Å
β = 129.724 (1)°
*V* = 2601.96 (14) Å^3^
*Z* = 4
Mo *K*α radiation
μ = 1.28 mm^−1^
*T* = 296 K
0.25 × 0.10 × 0.06 mm

#### Data collection

Bruker APEXII CCD diffractometer
11568 measured reflections
3129 independent reflections
2542 reflections with *I* > 2σ(*I*)
*R*_int_ = 0.033

#### Refinement

*R*[*F* ^2^ > 2σ(*F* ^2^)] = 0.030
*wR*(*F* ^2^) = 0.068
*S* = 1.03
3129 reflections
151 parameters
H-atom parameters constrained
Δρ_max_ = 0.35 e Å^−3^
Δρ_min_ = −0.29 e Å^−3^

### 

Data collection: *APEX2* (Bruker, 2007 ▶); cell refinement: *SAINT* (Bruker, 2007 ▶); data reduction: *SAINT*; program(s) used to solve structure: *SHELXS97* (Sheldrick, 2008 ▶); program(s) used to refine structure: *SHELXL97* (Sheldrick, 2008 ▶); molecular graphics: *ORTEP-3* (Farrugia, 1997 ▶); software used to prepare material for publication: *WinGX* (Farrugia, 1999 ▶) and *PLATON* (Spek, 2009 ▶).

## Supplementary Material

Crystal structure: contains datablocks global, I. DOI: 10.1107/S1600536810026668/hb5527sup1.cif

Structure factors: contains datablocks I. DOI: 10.1107/S1600536810026668/hb5527Isup2.hkl

Additional supplementary materials:  crystallographic information; 3D view; checkCIF report

## Figures and Tables

**Table 1:** Selected bond lengths (Å)

Ni1—S1	2.4708 (7)
Ni1—S2	2.4879 (5)
Ni1—S3	2.4995 (6)

**Table 2:** Hydrogen-bond geometry (Å, °)

*D*—H⋯*A*	*D*—H	H⋯*A*	*D*⋯*A*	*D*—H⋯*A*
N1—H1*A*⋯O1^i^	0.86	2.21	3.037 (4)	161
N1—H1*B*⋯O2	0.86	2.17	2.965 (3)	154
N2—H2*A*⋯O3^ii^	0.86	2.56	2.963 (3)	110
N2—H2*A*⋯O3^i^	0.86	2.30	3.110 (4)	157
N2—H2*B*⋯S2	0.86	2.63	3.449 (3)	159
N3—H3*A*⋯O3^iii^	0.86	2.19	3.022 (2)	163
N3—H3*B*⋯S1	0.86	2.72	3.5021 (19)	152
N3—H3*B*⋯S3	0.86	2.87	3.444 (2)	126
N4—H4*A*⋯O2^iii^	0.86	2.01	2.865 (3)	175
N4—H4*B*⋯S2^iv^	0.86	2.75	3.555 (2)	157
N5—H5*A*⋯S3^v^	0.86	2.82	3.623 (2)	155
N5—H5*A*⋯O1^vi^	0.86	2.48	2.922 (3)	113
N5—H5*B*⋯S1^vi^	0.86	2.59	3.410 (2)	160
N6—H6*A*⋯S2^vii^	0.86	2.83	3.464 (3)	132
N6—H6*A*⋯S3^v^	0.86	2.80	3.601 (2)	156
N6—H6*B*⋯O1^viii^	0.86	2.10	2.958 (3)	174

Symmetry codes: (i) 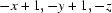; (ii) 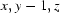; (iii) 
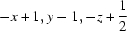
; (iv) 
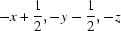
; (v) 
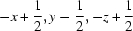
; (vi) 
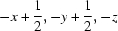
; (vii) 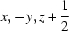; (viii) 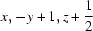.

**Fig. 1.**
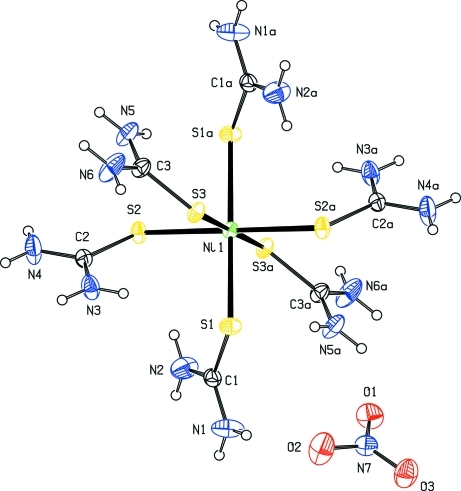
View of (I) with displacement ellipsoids depicted at the 30% probability level for all non-H atoms.

**Fig. 2.**
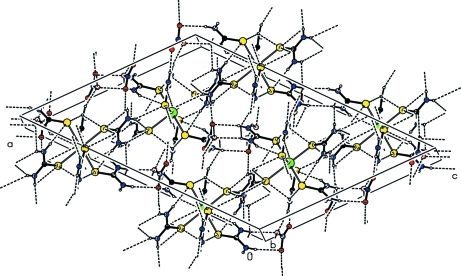
Partial view of the intra and intermolecular N—H···S and N—H···O hydrogen bonds in the crystal structure of (I), forming a three-dimensional network.

## supplementary crystallographic information

### Comment

The coordination chemistry of thiourea type ligands has been a matter of interest in view of their biological (Arslan *et al.*, 2009) and non-linear optical (Bhaskaran *et al.*, 2007) properties, and because of their potential use as selective reagents for concentration and separation of metal ions (Emre *et al.*, 2009). The complexes of nickel(II) with thioureas were shown to have a variety of stereochemistries (octahedral, tetragonal, square planar and tetrahedral) both in the solid state and in solution form (Eaton *et al.*, 1975; Figgis *et al.*, 1986; Suescun *et al.*, 2000; Zhu *et al.*, 2009). In order to investigate further about the structures of nickel(II)-thiourea systems, we present here a structural study of a Tu complex with nickel(II) nitrate, which consists of [Ni(Tu)_6_]^+2^ molecular ions and nitrate counter ions.

A preliminary X-ray study of complex (I) has been reported twice previously, but with incomplete crystallographic data (Maďar, 1961; Rodriguez *et al.*, 1961). We redetermined the crystal structure of complex (I), which we present in this paper.

In (I), the central Ni atom is located on a centre of inversion and is six-coordinated by six thiourea groups in a octahedral geometry (Fig. 1). The values of the geometrical parameters of the title molecule are as expected (Allen *et al.*, 1987). The Ni—S bond lengths vary from 2.4708 (7) to 2.4995 (6) Å.

In the crystal packing of (I), adjacent molecules are linked by intra and intermolecular N—H···S and N—H···O hydrogen bonds (Table 2, Fig. 2), forming a three-dimensional network and a supramolecular structure.

### Experimental

The complex was prepared by adding 4 equivalents of thiourea in 10 ml methanol to 1 mmole (0.29 g) solution of nickel(II) nitrate hexa hydrate in 10 ml methanol. After stirring the solution for half an hour, the green solution was filtered and the filtrate was kept for crystallization. As a result light green needles of (I) were formed.

### Refinement

H atoms were positioned geometrically and were treated as riding on their parent C atoms, with N—H = 0.86 Å and *U*_iso_(H) = 1.2*U*_eq_(N).

### Crystal data

**Table:**

[Ni(CH_4_N_2_S)_6_](NO_3_)_2_	*F*(000) = 1320
*M**_r_* = 639.50	*D*_x_ = 1.633 Mg m^−^^3^
Monoclinic, *C*2/*c*	Mo *K*α radiation, λ = 0.71073 Å
Hall symbol: -C 2yc	Cell parameters from 4823 reflections
*a* = 22.4433 (6) Å	θ = 2.5–28.2°
*b* = 9.2398 (3) Å	µ = 1.28 mm^−^^1^
*c* = 16.3136 (5) Å	*T* = 296 K
β = 129.724 (1)°	Needle, light green
*V* = 2601.96 (14) Å^3^	0.25 × 0.10 × 0.06 mm
*Z* = 4	

### Data collection

**Table:**

Bruker APEXII CCD diffractometer	2542 reflections with *I* > 2σ(*I*)
Radiation source: sealed tube	*R*_int_ = 0.033
graphite	θ_max_ = 28.3°, θ_min_ = 2.5°
φ and ω scans	*h* = −29→21
11568 measured reflections	*k* = −11→12
3129 independent reflections	*l* = −21→21

### Refinement

**Table:**

Refinement on *F*^2^	Primary atom site location: structure-invariant direct methods
Least-squares matrix: full	Secondary atom site location: difference Fourier map
*R*[*F*^2^ > 2σ(*F*^2^)] = 0.030	Hydrogen site location: inferred from neighbouring sites
*wR*(*F*^2^) = 0.068	H-atom parameters constrained
*S* = 1.03	*w* = 1/[σ^2^(*F*_o_^2^) + (0.0256*P*)^2^ + 1.7568*P*] where *P* = (*F*_o_^2^ + 2*F*_c_^2^)/3
3129 reflections	(Δ/σ)_max_ < 0.001
151 parameters	Δρ_max_ = 0.35 e Å^−^^3^
0 restraints	Δρ_min_ = −0.29 e Å^−^^3^

### Special details

**Table:**

Geometry. Bond distances, angles *etc*. have been calculated using the rounded fractional coordinates. All su's are estimated from the variances of the (full) variance-covariance matrix. The cell e.s.d.'s are taken into account in the estimation of distances, angles and torsion angles
Refinement. Refinement on *F*^2^ for ALL reflections except those flagged by the user for potential systematic errors. Weighted *R*-factors *wR* and all goodnesses of fit *S* are based on *F*^2^, conventional *R*-factors *R* are based on *F*, with *F* set to zero for negative *F*^2^. The observed criterion of *F*^2^ > σ(*F*^2^) is used only for calculating -*R*-factor-obs *etc*. and is not relevant to the choice of reflections for refinement. *R*-factors based on *F*^2^ are statistically about twice as large as those based on *F*, and *R*-factors based on ALL data will be even larger.

### Fractional atomic coordinates and isotropic or equivalent isotropic displacement parameters (Å^2^)

**Table:**

	*x*	*y*	*z*	*U*_iso_*/*U*_eq_	
Ni1	0.25000	0.25000	0.00000	0.0246 (1)	
S1	0.38321 (3)	0.34540 (6)	0.10478 (4)	0.0362 (2)	
S2	0.28084 (3)	−0.01160 (5)	0.01164 (4)	0.0331 (1)	
S3	0.28343 (3)	0.27376 (5)	0.17786 (4)	0.0311 (1)	
N1	0.49791 (11)	0.3572 (3)	0.10299 (17)	0.0630 (8)	
N2	0.42272 (13)	0.1592 (2)	0.02330 (19)	0.0630 (9)	
N3	0.40567 (10)	0.00546 (19)	0.21644 (13)	0.0475 (6)	
N4	0.36277 (12)	−0.2195 (2)	0.14917 (16)	0.0621 (7)	
N5	0.20704 (11)	0.0325 (2)	0.14718 (15)	0.0516 (7)	
N6	0.29045 (13)	0.1162 (2)	0.31643 (15)	0.0687 (8)	
C1	0.43826 (11)	0.2808 (2)	0.07398 (16)	0.0341 (6)	
C2	0.35527 (11)	−0.0788 (2)	0.13536 (15)	0.0324 (6)	
C3	0.25769 (12)	0.1293 (2)	0.21517 (16)	0.0363 (7)	
O1	0.41450 (9)	0.6847 (2)	−0.02032 (13)	0.0604 (6)	
O2	0.50573 (11)	0.6706 (2)	0.14802 (14)	0.0786 (7)	
O3	0.48990 (11)	0.86552 (19)	0.06598 (15)	0.0676 (7)	
N7	0.46952 (10)	0.7413 (2)	0.06486 (15)	0.0429 (6)	
H1A	0.52660	0.32720	0.08860	0.0940*	
H1B	0.50850	0.43750	0.13650	0.0940*	
H2A	0.45170	0.12990	0.00920	0.0940*	
H2B	0.38350	0.10840	0.00400	0.0940*	
H3A	0.44270	−0.03180	0.27720	0.0570*	
H3B	0.40160	0.09790	0.20880	0.0570*	
H4A	0.40020	−0.25490	0.21050	0.0930*	
H4B	0.33030	−0.27630	0.09690	0.0930*	
H5A	0.19560	−0.03860	0.16890	0.0620*	
H5B	0.18520	0.03990	0.08080	0.0620*	
H6A	0.27850	0.04460	0.33710	0.0820*	
H6B	0.32380	0.17910	0.36190	0.0820*	

### Atomic displacement parameters (Å^2^)

**Table:**

	*U*^11^	*U*^22^	*U*^33^	*U*^12^	*U*^13^	*U*^23^
Ni1	0.0289 (2)	0.0234 (2)	0.0217 (2)	−0.0015 (1)	0.0164 (1)	−0.0012 (1)
S1	0.0328 (2)	0.0410 (3)	0.0373 (3)	−0.0076 (2)	0.0235 (2)	−0.0106 (2)
S2	0.0400 (3)	0.0251 (2)	0.0238 (2)	0.0030 (2)	0.0156 (2)	0.0008 (2)
S3	0.0450 (3)	0.0272 (2)	0.0274 (2)	−0.0046 (2)	0.0260 (2)	−0.0013 (2)
N1	0.0430 (11)	0.0898 (17)	0.0669 (14)	−0.0207 (11)	0.0401 (11)	−0.0293 (12)
N2	0.0735 (14)	0.0459 (12)	0.1040 (18)	−0.0078 (11)	0.0727 (15)	−0.0195 (12)
N3	0.0421 (10)	0.0372 (10)	0.0320 (9)	0.0044 (8)	0.0093 (8)	0.0021 (8)
N4	0.0577 (13)	0.0333 (10)	0.0432 (12)	0.0039 (9)	0.0082 (10)	0.0092 (9)
N5	0.0610 (12)	0.0463 (11)	0.0399 (11)	−0.0225 (10)	0.0288 (10)	0.0010 (9)
N6	0.0915 (16)	0.0746 (15)	0.0338 (11)	−0.0326 (13)	0.0372 (12)	0.0040 (10)
C1	0.0305 (9)	0.0398 (12)	0.0301 (10)	0.0053 (8)	0.0185 (9)	0.0076 (9)
C2	0.0315 (9)	0.0325 (11)	0.0298 (10)	0.0033 (8)	0.0180 (8)	0.0039 (8)
C3	0.0413 (11)	0.0390 (12)	0.0309 (11)	−0.0049 (9)	0.0242 (9)	0.0030 (9)
O1	0.0486 (9)	0.0701 (12)	0.0348 (9)	−0.0114 (8)	0.0138 (8)	−0.0145 (8)
O2	0.0811 (13)	0.0679 (12)	0.0360 (10)	−0.0217 (11)	0.0139 (10)	−0.0029 (9)
O3	0.0798 (13)	0.0451 (11)	0.0654 (12)	−0.0100 (9)	0.0406 (11)	−0.0064 (9)
N7	0.0398 (10)	0.0498 (12)	0.0358 (10)	−0.0052 (9)	0.0226 (9)	−0.0074 (9)

### Geometric parameters (Å, °)

**Table:**

Ni1—S1	2.4708 (7)	N4—C2	1.312 (3)
Ni1—S2	2.4879 (5)	N5—C3	1.308 (3)
Ni1—S3	2.4995 (6)	N6—C3	1.316 (3)
Ni1—S1^i^	2.4708 (7)	N1—H1B	0.8600
Ni1—S2^i^	2.4879 (5)	N1—H1A	0.8600
Ni1—S3^i^	2.4995 (6)	N2—H2A	0.8600
S1—C1	1.711 (3)	N2—H2B	0.8600
S2—C2	1.715 (2)	N3—H3A	0.8600
S3—C3	1.713 (2)	N3—H3B	0.8600
O1—N7	1.239 (3)	N4—H4B	0.8600
O2—N7	1.232 (3)	N4—H4A	0.8600
O3—N7	1.231 (3)	N5—H5A	0.8600
N1—C1	1.306 (4)	N5—H5B	0.8600
N2—C1	1.304 (3)	N6—H6A	0.8600
N3—C2	1.313 (3)	N6—H6B	0.8600
			
S1—Ni1—S2	98.00 (2)	C2—N3—H3B	120.00
S1—Ni1—S3	80.37 (2)	C2—N3—H3A	120.00
S1—Ni1—S1^i^	180.00	H3A—N3—H3B	120.00
S1—Ni1—S2^i^	82.00 (2)	C2—N4—H4B	120.00
S1—Ni1—S3^i^	99.63 (2)	C2—N4—H4A	120.00
S2—Ni1—S3	97.72 (2)	H4A—N4—H4B	120.00
S1^i^—Ni1—S2	82.00 (2)	H5A—N5—H5B	120.00
S2—Ni1—S2^i^	180.00	C3—N5—H5A	120.00
S2—Ni1—S3^i^	82.28 (2)	C3—N5—H5B	120.00
S1^i^—Ni1—S3	99.63 (2)	C3—N6—H6B	120.00
S2^i^—Ni1—S3	82.28 (2)	H6A—N6—H6B	120.00
S3—Ni1—S3^i^	180.00	C3—N6—H6A	120.00
S1^i^—Ni1—S2^i^	98.00 (2)	O2—N7—O3	120.0 (2)
S1^i^—Ni1—S3^i^	80.37 (2)	O1—N7—O2	119.6 (2)
S2^i^—Ni1—S3^i^	97.72 (2)	O1—N7—O3	120.29 (19)
Ni1—S1—C1	116.99 (8)	S1—C1—N2	122.4 (2)
Ni1—S2—C2	117.12 (7)	S1—C1—N1	118.06 (18)
Ni1—S3—C3	115.14 (7)	N1—C1—N2	119.5 (3)
C1—N1—H1B	120.00	S2—C2—N4	118.85 (16)
H1A—N1—H1B	120.00	S2—C2—N3	122.36 (15)
C1—N1—H1A	120.00	N3—C2—N4	118.78 (19)
H2A—N2—H2B	120.00	N5—C3—N6	119.2 (2)
C1—N2—H2A	120.00	S3—C3—N5	122.62 (17)
C1—N2—H2B	120.00	S3—C3—N6	118.22 (17)
			
S2—Ni1—S1—C1	47.38 (8)	S2—Ni1—S3—C3	−38.66 (11)
S3—Ni1—S1—C1	143.91 (8)	S1^i^—Ni1—S3—C3	44.48 (11)
S2^i^—Ni1—S1—C1	−132.62 (8)	S2^i^—Ni1—S3—C3	141.34 (11)
S3^i^—Ni1—S1—C1	−36.09 (8)	Ni1—S1—C1—N1	159.63 (16)
S1—Ni1—S2—C2	48.29 (12)	Ni1—S1—C1—N2	−20.5 (2)
S3—Ni1—S2—C2	−33.00 (12)	Ni1—S2—C2—N3	−15.4 (3)
S1^i^—Ni1—S2—C2	−131.71 (12)	Ni1—S2—C2—N4	165.6 (2)
S3^i^—Ni1—S2—C2	147.00 (12)	Ni1—S3—C3—N5	−17.6 (3)
S1—Ni1—S3—C3	−135.52 (11)	Ni1—S3—C3—N6	162.5 (2)

Symmetry codes: (i) −*x*+1/2, −*y*+1/2, −*z*.

### Hydrogen-bond geometry (Å, °)

**Table:**

*D*—H···*A*	*D*—H	H···*A*	*D*···*A*	*D*—H···*A*
N1—H1A···O1^ii^	0.86	2.21	3.037 (4)	161
N1—H1B···O2	0.86	2.17	2.965 (3)	154
N2—H2A···O3^iii^	0.86	2.56	2.963 (3)	110
N2—H2A···O3^ii^	0.86	2.30	3.110 (4)	157
N2—H2B···S2	0.86	2.63	3.449 (3)	159
N3—H3A···O3^iv^	0.86	2.19	3.022 (2)	163
N3—H3B···S1	0.86	2.72	3.5021 (19)	152
N3—H3B···S3	0.86	2.87	3.444 (2)	126
N4—H4A···O2^iv^	0.86	2.01	2.865 (3)	175
N4—H4B···S2^v^	0.86	2.75	3.555 (2)	157
N5—H5A···S3^vi^	0.86	2.82	3.623 (2)	155
N5—H5A···O1^i^	0.86	2.48	2.922 (3)	113
N5—H5B···S1^i^	0.86	2.59	3.410 (2)	160
N6—H6A···S2^vii^	0.86	2.83	3.464 (3)	132
N6—H6A···S3^vi^	0.86	2.80	3.601 (2)	156
N6—H6B···O1^viii^	0.86	2.10	2.958 (3)	174

Symmetry codes: (ii) −*x*+1, −*y*+1, −*z*; (iii) *x*, *y*−1, *z*; (iv) −*x*+1, *y*−1, −*z*+1/2; (v) −*x*+1/2, −*y*−1/2, −*z*; (vi) −*x*+1/2, *y*−1/2, −*z*+1/2; (i) −*x*+1/2, −*y*+1/2, −*z*; (vii) *x*, −*y*, *z*+1/2; (viii) *x*, −*y*+1, *z*+1/2.
